# ZrO_2_-TiO_2_ Incorporated PVDF Dual-Layer Hollow Fiber Membrane for Oily Wastewater Treatment: Effect of Air Gap

**DOI:** 10.3390/membranes10060124

**Published:** 2020-06-16

**Authors:** Nurshahnawal Yaacob, Pei Sean Goh, Ahmad Fauzi Ismail, Noor Aina Mohd Nazri, Be Cheer Ng, Muhammad Nizam Zainal Abidin, Lukka Thuyavan Yogarathinam

**Affiliations:** 1 Advanced Membrane Technology Research Centre (AMTEC), School of Chemical and Energy Engineering, Universiti Teknologi Malaysia, 81300 Skudai, Johor, Malaysia; nurshahnawal@unikl.edu.my (N.Y.); peisean@petroleum.utm.my (P.S.G.); ngbecheer@petroleum.utm.my (B.C.N.); m.nizam@utm.my (M.N.Z.A.); lukkathuyavan@gmail.com (L.T.Y.); 2 Malaysian Institute of Marine Engineering Technology (MIMET), Universiti Kuala Lumpur, 32200 Lumut, Perak, Malaysia; 3 Malaysian Institute of Chemical and Bio–Engineering Technology (MICET), Universiti Kuala Lumpur, 78000 Alor Gajah, Melaka, Malaysia; nooraina@unikl.edu.my

**Keywords:** air gap, hollow fiber spinning, dual-layer hollow fiber, oily wastewater treatment

## Abstract

Dual-layer hollow fiber (DLHF) nanocomposite membrane prepared by co-extrusion technique allows a uniform distribution of nanoparticles within the membrane outer layer to enhance the membrane performance. The effects of spinning parameters especially the air gap on the physico-chemical properties of ZrO_2_-TiO_2_ nanoparticles incorporated PVDF DLHF membranes for oily wastewater treatment have been investigated in this study. The zeta potential of the nanoparticles was measured to be around –16.5 mV. FESEM–EDX verified the uniform distribution of Ti, Zr, and O elements throughout the nanoparticle sample and the TEM images showed an average nanoparticles grain size of ~12 nm. Meanwhile, the size distribution intensity was around 716 nm. A lower air gap was found to suppress the macrovoid growth which resulted in the formation of thin outer layer incorporated with nanoparticles. The improvement in the separation performance of PVDF DLHF membranes embedded with ZrO_2_-TiO_2_ nanoparticles by about 5.7% in comparison to the neat membrane disclosed that the incorporation of ZrO_2_-TiO_2_ nanoparticles make them potentially useful for oily wastewater treatment.

## 1. Introduction

Oily wastewaters are normally released to sea without proper treatment and are above the allowable standard discharge limits [1]. The directly discharged oily wastewaters has contributed to major contamination in the seawater as well as disturbed the ecosystems of marine creatures and human wellbeing [2].

Membrane technology has emerged as an eminent technology in oily wastewater treatment due to its high separation efficiency [3], high effluent quality [4], and no chemical additive is needed to break the emulsion. Microfiltration (MF), ultrafiltration (UF), nanofiltration (NF), and pervaporation (PV) membranes are frequently employed to remove oil-water emulsion [5] owing to the high efficacy of the membranes in the removal of oil droplets [6]. However, the high fouling propensity resulted from oil adsorption and deposition on the surface of these polymeric membranes has a negative impact on water permeability hence limited their usage at the industry level [7,8].

Many researchers devoted their research to investigate ways to boost the antifouling properties of polymeric membranes. Modification of polymer membrane surface through various strategies have been accomplished to tailor the surface hydrophilicity, charge, and roughness of the membrane in order to mitigate the effects of fouling. The incorporation of nanoparticles such as hydrophilic TiO_2_ [6,9,10,11,12], hydrous manganese [8], hydrous aluminum oxide [13], graphene oxide [14], and SiO_2_-g-PEGMA [3] into both flat-sheet and hollow fiber UF membranes to treat oily wastewater has endowed improved membrane surface hydrophilicity and antifouling properties. Ong et al. observed that although the good antifouling property of polyvinylidene fluoride (PVDF)-TiO_2_ composite hollow fiber membrane was compromised when it was tested at high concentration of oily solution of 1000 ppm [9], the composite membrane was good enough to treat discharged containing oily solution from industries without having to suffer severe flux declination since the concentration of oil that originates from industrial effluent normally falls in the range of 100 to 450 ppm.

TiO_2_ is one of the most studied nanomaterials for membrane modifications [15] due to its availability, notable physical and chemical properties, and antifouling potential [16]. The properties of TiO_2_ can be further enhanced through metal–ion doping to improve the hydrophilicity of the nanoparticles [17]. Most of the polymeric hollow fiber membrane immobilized with nanoparticles belong to mixed matrix nanocomposite membrane where the inorganic nanoparticles are randomly distributed throughout the polymer matrix [18,19]. As such, the incorporation of inorganic nanoparticles such as metal oxides, zeolites, metal-organic frameworks, and carbon nanotubes resulted in remarkable separation performance but still facing problems related to aging and fouling [20].

Yu et al. [21] integrated TiO_2_ into UF hollow fiber membranes and observed increased membrane permeability with the addition of 1 wt% TiO_2_. Although TiO_2_ is recognized as prevalent inorganic materials with renowned chemical and physical stability, TiO_2_ is prone to go through phase transformation from anatase to rutile. To overcome this problem, the addition of ZrO_2_ was introduced into TiO_2_ to suppress the phase transformation [22]. The addition of ZrO_2_ not only assisted in stabilizing the active phase in a finely dispersed state but also improved the surface area of the nanoparticles [23]. Improvement in membrane permeability and control of membrane–surface properties have also been reported with the addition of hydrophilic ZrO_2_ nanoparticles [24]. Sotto et al. [25] reported flux and antifouling properties enhancement in TiO_2_-ZrO_2_ polyethersulfone UF flat sheet membranes. Water flux of about 215 L/m^2^·h and bovine serum albumin rejection of 96% were reported. Later, Khan et al. [26] evaluated the membrane fouling performance of TiO_2_-ZrO_2_ incorporated in PVDF MF hollow fiber membranes for humic acid treatment. The study reported total organic carbon removal of about 30% when the permeate was steadily withdrawn at 100 L/m^2^·h of fixed flux. So far, no study on ZrO_2_-TiO_2_ UF hollow fiber membranes has been reported.

On the other hand, dual-layer hollow fiber (DLHF) membranes have attracted great interest due to its potential to improve water flux, reduce material cost through the usage of less costly material as substrate, and improve antifouling performance through the addition of nanoparticles [27,28,29]. The presence of immobilized TiO_2_ nanoparticles in the PVDF outer layer of the DLHF membrane promoted the formation of microporous structure which allowed uniform nanoparticles dispersion [30,31].

Despite the growing number of experimental studies on DLHF membranes [31,32,33,34], no study on the effect of a spinning parameter such as the air gap during dry-wet spinning has been reported. Most of the works related to the effect of the air gap were focused on single-layer [35,36,37] and mixed matrix hollow fiber membranes [38]. The fine-tuning of the air gap during the dry-wet hollow fiber spinning is crucial to produce a membrane with good structural integrity and lack of delamination between layers [38]. A proper control of air gap based on the characteristics of the polymers could tailor the cross-sectional morphology of the resultant membrane, hence achieving better separation.

Khayet et al. [35] stated that the air gap created elongational stress as a result of gravity on the internal or external surfaces of PVDF hollow fibers. A thinner skin layer of asymmetric PVDF membranes could be obtained when spun at a lower air gap with water used as an internal and external coagulant. At a lower air gap, the inner surface help in controlling the UF performance of the PVDF hollow fiber membranes through pore size reduction and tighter molecular packing.

Korminouri et al. [36] reported that the air gap increments from 0 to 50 cm in surface-modified polysulfone hollow fiber membrane resulted in the enhancement of the membrane surface hydrophobicity. Fang et al. [37] observed the enhancement in membrane water permeation from 266 to 1429 L/m^2^·h·bar when the air gap was increased from 0.5 to 20 cm. Mubashir et al. [38] reported the reduction in the outer diameters of cellulose acetate hollow fiber mixed matrix membranes (HFMMMs) from 719.1 µm to 648.4 µm with air gap increment from 2.5 to 7.5 cm. The optimum air gap length of 5.0 cm exhibited a reduction in the growth of macrovoids in HFMMMs due to the increment of residence time.

The closest work on DLHF membrane was presented by Le et al. [39]. A dense outer layer structure with no macrovoids was observed when the membrane was spun at the air gap of 3 cm. The inner layer exhibited both finger-like and sponge-like structure. The finger-like macrovoids act as permeate transport channels to the lumen side of the hollow fibers whereas the sponge-like structure provided mechanical support. The membranes exhibited a flux of 1.9 kg/m^2^·h and ethanol dehydration with a comparable separation factor of 166. Later, Dzinun et al. [30] spun DLHF membrane immobilized with TiO_2_ at an air gap length of 10 cm and observed the formation of a finger-like structure at both outer and inner layer and a sponge-like structure formed at the intermediate layer.

Based on the above discussion, optimization of the spinning parameters of DLHF membrane incorporated with nanoparticles is worth pursuing. Thus, this study aimed to optimize the air gap length of the PVDF DLHF membrane incorporated with 1 wt% ZrO_2_-TiO_2_ nanoparticles in the selective layer for oily wastewater treatment. The oil removal efficiency and filtration efficacy are used to assess the membrane performance. The nanoparticles were characterized using dynamic light scattering, FESEM, EDX, and TEM. Meanwhile, the morphology, wettability, and porosity of the DLHF membranes were characterized using SEM, water contact angle goniometer, and porosity measurement, respectively.

## 2. Materials and Methods

### 2.1. Materials

For the synthesis of nanoparticles, titanium (IV) isopropoxide 97% (TTIP, Sigma–Aldrich, St. Louis, MO, USA), and tetrapropyl zirconate (TPZ, Sigma–Aldrich) were employed as the precursors for TiO_2_ and ZrO_2_. Technical grade sodium dodecylbenzenesulfonate (SDBS, Sigma–Aldrich) was used as surfactant for oily wastewater. Isopropyl alcohol (IPA, Sigma–Aldrich) was used as solvent. Nitric acid 70% (RCl Labscan Limited, Bangkok, Thailand) was used as oxidizing agent.

For membrane fabrication, polyvinylidene fluoride (PVDF Kynar 760 pellet, Arkema Inc., Philadelphia, PA, USA) was used as membrane material for both inner and outer dope solution. The PVDF pellets were dried in a vacuum oven at 60 °C for 24 h. 1–Methyl–2–pyrrolidone (NMP, Merck Millipore, Burlington, MA, USA) was used as received as solvent to prepare dope solution and polyvinylpyrrolidone (PVP, UNI–CHEM, Crnotravska, Beograd, Serbia) was used as pore former. Industrial glycerol was used during the hollow fiber post-treatment.

For synthetic oily wastewater preparation, the commercial premium Nu-Clear thread cutting oil #70835 (RIDGID, Ridge Tool Company, Elyria, OH, USA) was mixed with deionized (D.I.) water and SDBS.

### 2.2. Synthesis of Nanoparticles

ZrO_2_-TiO_2_ nanoparticles were synthesized using sol–gel method as described elsewhere [40] using TTIP and TPZ as the precursors for TiO_2_ and ZrO_2_, respectively. The mixture of 9.9 mL of TTIP (9.504 g, 0.033 mol) and 0.1 mL of TPZ (0.1044 g, 0.00032 mol) in 90 mL of IPA was added dropwise into 900 mL of D.I. water that was maintained at the pH of 1.5 using nitric acid under constant stirring. The temperature of the reaction tub was maintained at 2 °C throughout the stirring until a colloidal sol was obtained. After the aging period of 24 h at room temperature, the colloidal sol was precipitated through evaporative drying at 90 °C until the colloidal sol dried and formed crystals. The resulting nanoparticles were then dried in vacuum oven at 105 °C for 4 h and later calcined at 500 °C in furnace (Nabertherm GmbH, Lilienthal, Germany) with air flowing continuously for 3 h to remove the solvents [41].

### 2.3. Preparation of PVDF DLHF

**Figure 1:**
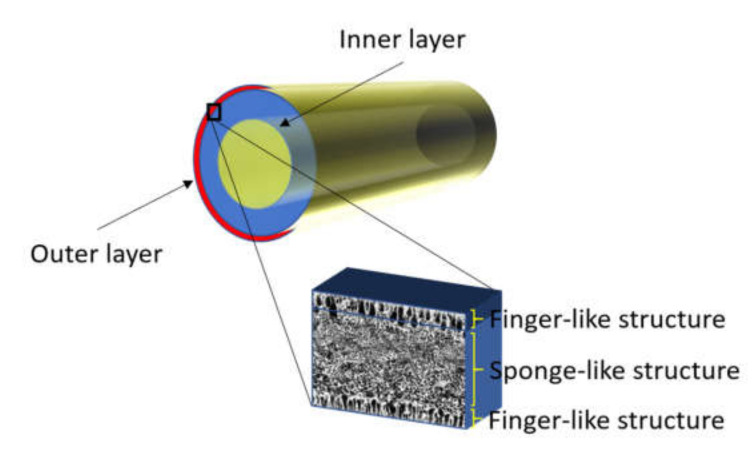
Schematic diagram of the dual-layer hollow fiber (DLHF) membrane structure with ZrO_2_-TiO_2_ embedded polyvinylidene fluoride (PVDF) outer layer and PVDF inner layer.

For inner dope solution, 5 wt% of PVP was mixed into 77 wt% of NMP solvent in a Schott bottle and stirred with an overhead stirrer at 450 rpm until all PVP was completely dissolved. Then, 18 wt% of PVDF in pellet form were put into the dope solution and mixed to form a homogeneous solution. The outer dope solution composition was kept constant at PVDF/NMP/PVP of 15 wt%/80 wt%/5 wt% [30]. The concentration of PVDF in the outer dope was less than that of the inner dope mixture to cater for the addition of nanoparticles. In this study, the composition for the outer dope solution containing ZrO_2_-TiO_2_ nanoparticles was fixed at 1 wt%. Based on preliminary study, 1 wt% ZrO_2_-TiO_2_ nanoparticles were found to exhibit a high specific surface area. The spinning parameters used to fabricate the PVDF/ZrO_2_-TiO_2_ DLHF membranes are summarized in Table 1. The inner dope flowrate was selected based on the previous study [42]. The ZrO_2_-TiO_2_ nanoparticles loading percentage was determined by the total weight of the neat membrane dope solution, which is fixed at 100% (i.e., 1% ZrO_2_-TiO_2_ nanoparticles of 100 g would be 1 g of ZrO_2_-TiO_2_ nanoparticles into 100 g of dope solution formula). The neat and 1 wt% of ZrO_2_-TiO_2_ membranes prepared are denoted as DL-ZT0 and DL-ZT1, respectively. Neat DLHF membrane was produced with no added ZrO_2_-TiO_2_ nanoparticles to investigate the effect of air gap length on membrane performance and characteristics. The DLHF membrane structure is shown in Figure 1.

The dope solution was degassed in an ultrasonic bath (Symphony VWR Ultrasonic Cleaner, Avantor, Radnor, PA, USA) prior to spinning. The hollow fiber membrane was spun using triple orifice spinneret with a hole size dimension of 1.2 mm/0.8 mm/0.4 mm to extrude the dope solution to form DLHF membranes with dope extrusion rate of 4 rpm. The air gap was adjusted in the range of 5 to 50 cm. Water was used as bore fluid and external coagulant. The produced membrane was then soaked in water for 1 day to ensure complete solvent residue removal. The membrane was immersed into 10 wt% glycerol aqueous solution for 24 h to improve the integrity of the membrane. The membranes were left to dry at room temperature for 48 h prior to module fabrication and membrane characterization.

### 2.4. Characterizations

For the nanoparticles, the particle size distribution and zeta potential in suspension were evaluated by dynamic light scattering (Litesizer 500 Anton Paar, Ireland, UK) at 25 °C. The nanoparticle powder was manually crushed and sieved before diluted with distilled water. The zeta potential was calculated using the Smoluchowski approximation and electrophoretic mobility. A deconvolution process using Lorentz curves was performed in Excel by Solver. A Zeiss Crossbeam 340 field emission scanning electron microscopy (FESEM, Oberkochen, Germany) equipped with an Oxford instrument energy-dispersive x-ray (EDX, Abingdon, UK) was employed for elemental and chemical analysis. The non-conductive nanoparticles were coated with an additional thin layer (~10 nm) of gold to remove the charging effect. Transmission electron microscopy (TEM, HT7700, Hitachi, Fukuoka, Japan) was used to inspect the nanostructure of the synthesized nanoparticles.

For the membranes, a scanning electron microscope (SEM, TM3000 Hitachi High Technologies America, Schaumburg, IL, USA) was employed to investigate the outside surface and cross-section structure of the hollow fiber membranes. The defect-free hollow fiber samples were cut into small pieces and fractured in liquid nitrogen. The sample attached to the aluminum stub was then sputtered with platinum under vacuum for 3 min using SC7620 ‘Mini’ sputter coater/glow discharge system (Quorum Technologies, Laughton, East Sussex, UK). The membrane contact angle test was done via sessile drop technique using D.I. water to determine the hydrophilicity and wettability of the membrane. The experiment was conducted on a Goniometer (Model: OCA, Dataphysics, Filderstadt, Germany) and the static angle was determined using a computer-controlled automatic liquid deposition system (Software: SCA 20, Dataphysics, Filderstadt, Germany). The contact angles were measured at ambient temperature. Three membrane samples of length 5 cm were cut and properly secured on the hollow fiber membrane holder. The microsyringe was filled with D.I. water and installed at the electronic syringe module system. The needle was then aligned above the specimen surface. The gap between the needle and the fiber was estimated to ensure 0.5 µl of dosing volume released at a dosing rate of 1 µL/s from the needle on top of the specimen surface. The moving platform was moved upward to bring the fiber into contact with the drop produced from the needle tip. Moving the platform slowly downward produced a droplet with base attached and spread on the fiber. The contact angle was then measured before the fiber was moved to a new position. 15 to 20 multi-point readings were taken, and the contact angle readings were averaged to improve accuracy. The membrane porosity, ε, is determined from

$$\varepsilon =\frac{\frac{w_{wet}-w_{dry}}{\rho_{w}}}{\frac{w_{wet}-w_{dry}}{\rho_{w}}+\frac{w_{dry}}{\rho_{p}}}\times 100$$

where *ε* is the membrane porosity (%), *w_wet_* is the weight of wet membrane (g), *w_dry_* is the weight of dry membrane (g), *ρ_p_* is the density of the polymer (g/cm^3^), and *ρ_w_* is the density of water (g/cm^3^).

### 2.5. Membrane Performance Evaluation

Pure water flux of the hollow fiber membrane was evaluated at a constant temperature of 25 °C using crossflow filtration system in three modules each containing five fibers with one end sealed. Prior to the test, the hollow fiber membranes were soaked in tap water for 30 min and followed by a pre-compression at 1 bar pressure for another 30 min to attain stable pure water volume flux. The pure water flux, *J_w_* was determined using Equation (1) [43]:

*J_w_* = *Q*/(*A*·*t*), (1)

where *J_w_* signifies the permeation flux in L/m^2^·h. *Q* represents the amount of permeated pure water in L. *A* corresponds to the effective area of the membrane in m^2^ and *t* is the time required to get the amount of *Q* in hour. The pure water permeability (PWP) was calculated by using Equation (2) [14]. TMP is the transmembrane pressure in bar.

PWP = *J_w_*/TMP, (2)

The membrane oil rejection percentage (*R*) was determined based on Equation (3) [44]. The oil concentrations in the permeate and feed were determined using PerkinElmer UV-Vis Spectrometer Lambda 25 (Waltham, MA, USA) at the maximum absorbance of oily wastewater measured at 223 nm. *C_P_* and *C_F_* signify the permeate and the feed concentration in ppm, respectively.

*R* = (1 − (*C_P_*/*C_F_*)) × 100, (3)

### 2.6. Preparation of Synthetic Oily Wastewater

The oily wastewater model pollutant was prepared by mixing 9 parts of the pre-mixed commercial cutting oil with 1 part of SDBS in a high-speed blender (Model: BL 310AW, Khind, Shah Alam, Malaysia) within the surrounding environment at the speed of 50 Hz for about 2 min as described elsewhere [45]. The desired amount of cutting oil and SDBS were mixed with 1 l of D.I. water to prepare synthetic oily wastewater with concentrations of 1000 ppm.

## 3. Results and Discussion

### 3.1. Surface Charge Analysis

**Figure 2:**
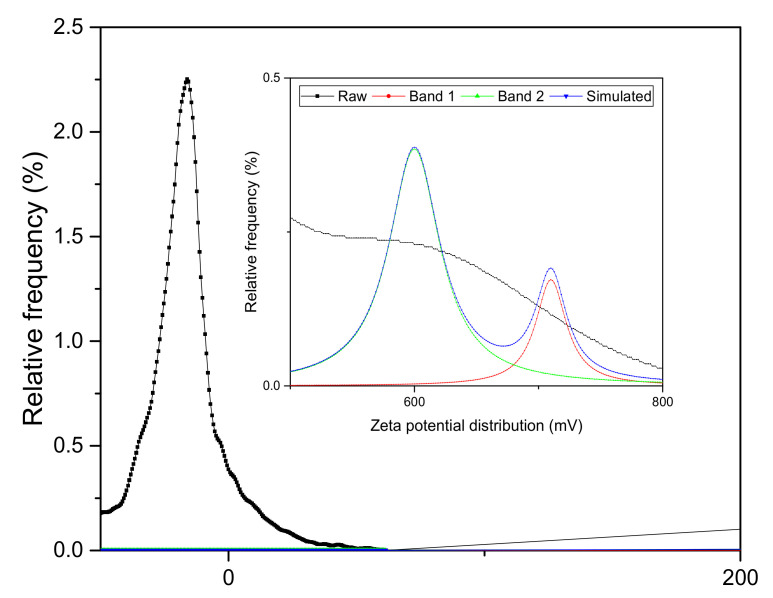
Zeta potential of ZrO_2_-TiO_2_ nanoparticles with inset showing the deconvolution of two peaks.

The zeta potential of the nanoparticles was –16.5 mV as shown in Figure 2. The nanoparticles with the above feature are subjected to low stability of the colloidal suspension which was expected to be based on electrostatic considerations. In general, there is a probability of particle aggregation that might affect suspension stability in the dispersion medium [46,47,48]. Two deconvolution peaks were observed at 600 mV and 710 mV.

### 3.2. Nanostructure and Elemental Composition Analysis

**Figure 3:**
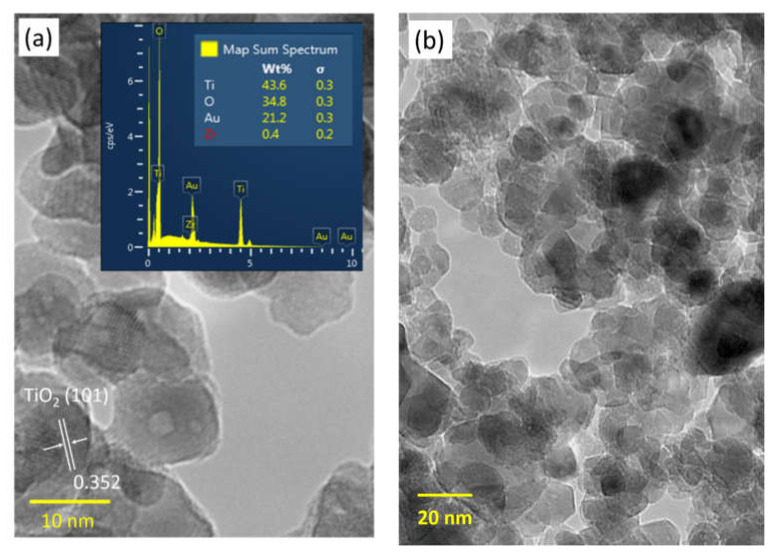
Transmission electron microscopy (TEM) images of ZrO_2_-TiO_2_ nanoparticles. (**a**) TiO_2_ crystal spacing with inset shows the peaks and the percentage of elemental composition and (**b**) Small agglomeration and dispersion of nanoparticles.

**Figure 4:**
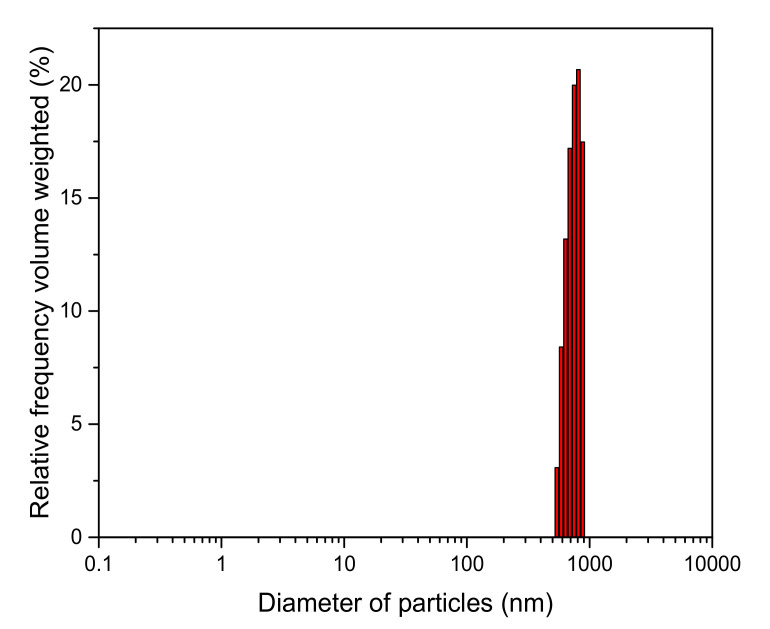
Distribution of ZrO_2_-TiO_2_ nanoparticles

The microstructure of the ZrO_2_-TiO_2_ nanoparticles with the average grain size of the nanoparticles around 12 nm can be observed from TEM images as shown in Figure 3a. The interplanar crystal spacing of 0.352 nm corresponds to the (1 0 1) plane of anatase TiO_2_. The peaks corresponding to Ti, Zr, and O elements were clearly observed at their normal energy levels, showing the elemental composition, atomic percentage, and weight percentage of the nanoparticles without the presence of any impurities. The presence of Au was due to the gold sputtering. It was observed that Ti, Zr, and O elements were uniformly distributed throughout the sample. This result also verified that ZrO_2_ doped TiO_2_ was successfully prepared via sol–gel method. Meanwhile, the size distribution of the 1 wt% ZrO_2_-TiO_2_ nanoparticles was 716 nm as shown in Figure 4, which confirmed that the particle was in the nano-size range.

### 3.3. Effect of Air Gap Length on Membrane Pore Size and Morphology

**Figure 5:**
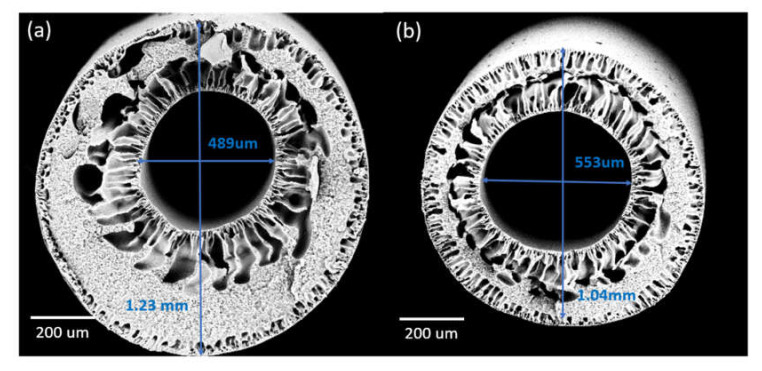
The overall (×100 magnification) structure of DLHF membranes spun at air gap of 5 cm for (**a**) DL-ZT0 and (**b**) DL-ZT1.

Varying the air gap during spinning could impart significant effects on the membrane pore sizes and morphologies. Increasing the air gap from 5 to 50 cm in the DLHF membranes embedded with ZrO_2_-TiO_2_ nanoparticles (DL-ZT1) resulted in the shrinkage of the fiber diameter dimensions of the outer layer but the expansion of the inner layer diameter (at air gap of 5 and 10 cm only) in comparison to the neat membranes (DL-ZT0). Figure 5a,b showed the diameter of the DLHF spun at an air gap of 5 cm which was directly measured from the SEM images. The finger-like structure developed at both inner and outer layers. An increase in the air gap caused the outer layer to become denser while the finger-like structure became smaller and shorter.

**Figure 6:**
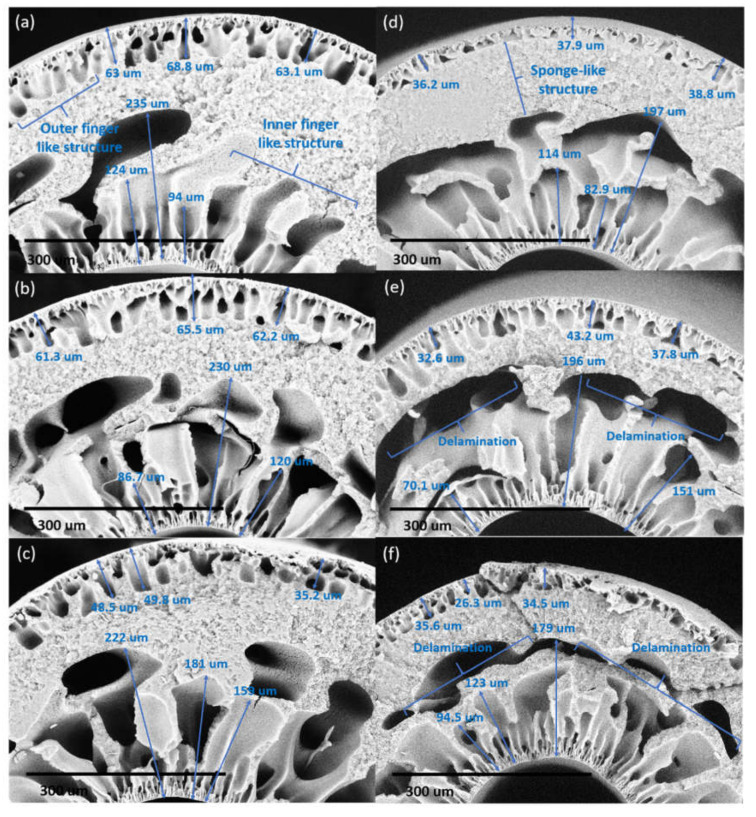
The cross-section (×300 magnification) structure of DL-ZT0 membranes spun at different air gap of (**a**) 5 cm, (**b**) 10 cm, (**c**) 20 cm, (**d**) 30 cm, (**e**) 40 cm, and (**f**) 50 cm.

**Figure 7:**
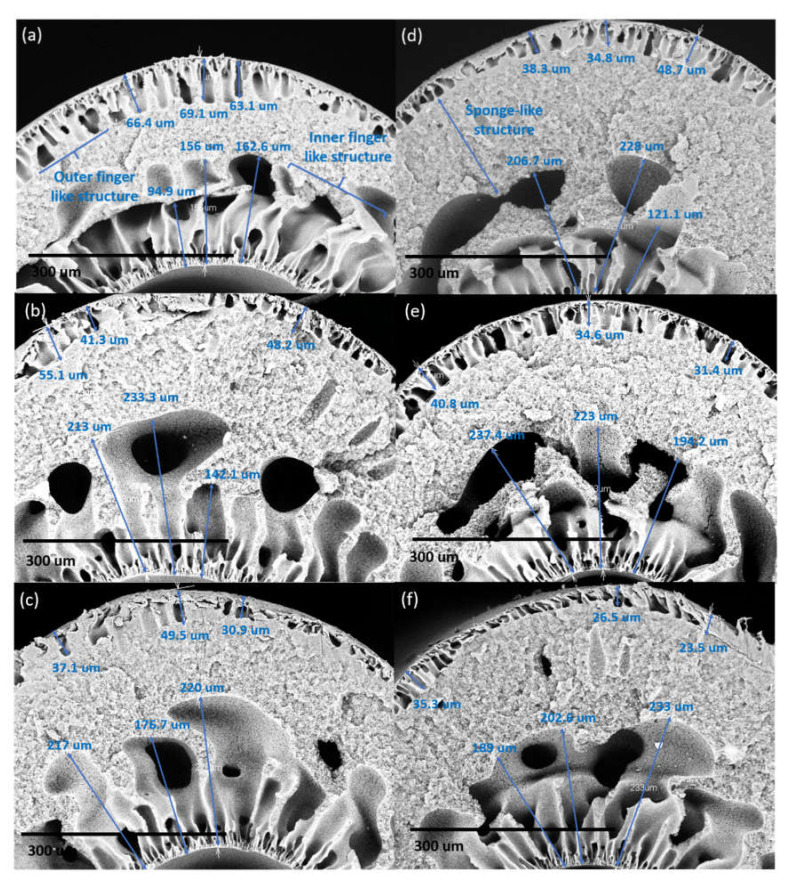
The cross-section (×300 magnification) structure of DL-ZT1 membranes spun at different air gap of (**a**) 5 cm, (**b**) 10 cm, (**c**) 20 cm, (**d**) 30 cm, (**e**) 40 cm, and (**f**) 50 cm.

The length of the finger-like structure at the outer layer of DL-ZT0 as shown in Figure 6a–f was measured as 65.0 ± 3.3, 63.0 ± 2.2, 44.5 ± 8.1, 37.6 ± 1.3, 37.9 ± 5.3 and 32.1 ± 5.1 µm for the air gap length of 5 cm (DL-ZT0-05), 10 cm (DL-ZT0-10), 20 cm (DL-ZT0-20), 30 cm (DL-ZT0-30), 40 cm(DL-ZT0-40), and 50 cm (DL-ZT0-50), respectively. Meanwhile, the length of the finger-like structure at the outer layer of DL-ZT1 as shown in Figure 7a–f showed a similar trend of reduction in the finger-like size measured as 66.2 ± 3.0, 48.2 ± 6.9, 39.2 ± 9.5, 40.6 ± 7.2, 35.6 ± 4.8 and 28.4±6.1 µm at similar air gap of 5 cm (DL-ZT1-05), 10 cm (DL-ZT1-10), 20 cm (DL-ZT1-20), 30 cm (DL-ZT1-30), 40 cm (DL-ZT1-40) and 50 cm (DL-ZT1-50), respectively. The finger-like structure at the outer layer of DL-ZT0 (except DL-ZT0-05 and DL-ZT0-30) is longer than that of DL-ZT1. On the other hand, the non-uniform finger-like structure at the inner layer of DL-ZT0 decreased from the average of 151 to 132 µm when the air gap was increased from 5 to 50 cm. However, an opposite trend was observed in DL-ZT1 with an increasing finger-like size from the average of 138 to 208 µm.

The air gap length affected the formation of the finger-like at the outer and inner layers in DL-ZT0. The finger-like at the outer layer showed a reducing trend when the air gap length was increased. The outer layer of the hollow fiber was in contact with air medium before contact with water. This has caused the surface of the outer layer to solidify in air during the traveling, hence prevented the immediate contact between the polymer solution of the outer layer with the water quenching bath hence slowing down the phase inversion at the outer surface. However, at a lower air gap, the short contact time between the polymer and the water quenching bath allowed immediate phase inversion and subsequent solidification to be initiated from the outer surface and proceeded inward thus increased the finger-like structure at the outer layer [37].

On the other hand, the finger-like structure at the inner layer showed the opposite trend whereby the finger-like structure was much bigger in size when the hollow fiber was spun with a larger air gap. This was due to the fast phase inversion by the bore fluid since the inner layer was already in contact with the bore fluid inside the spinneret from the moment the inner layer was established. The increase in the finger-like structure size was due to the longer contact time of the spun fiber with the inner coagulant [36]. In DL-ZT1, the formation of finger-like structure on the outer surface was longer in DL-ZT1-05 and DL-ZT1-30 while showing reducing trend in DL-ZT1-10, DL-ZT1-20, DL-ZT1-40 and DL-ZT1-50. This might be due to the presence of ZrO_2_-TiO_2_ nanoparticles which has improved the membrane hydrophilicity and promoted the growing of microvoids at the outer layer upon the contact with the water of the coagulation bath [49,50]. Despite the tendency of ZrO_2_-TiO_2_ nanoparticles to agglomerate [22], the reduction in particle size of the binary metal oxide has resulted in enhanced nanoparticles dispersity of the agglomerate formed. The incorporation of ZrO_2_-TiO_2_ nanoparticles at low amount has been reported to form narrow microvoids with thin skin layer [25]. TEM images in Figure 3b showed the agglomeration of small grains and some dispersed nanoparticles that was due to the overlapping of some bigger particles with the smaller particles [51].

The sponge-like structure was sandwiched between the outer and inner finger-like layer due to the instability of the suspension–coagulant interface during the phase inversion process [52]. The solvent–nonsolvent exchange rate during immersion precipitation occurred through a delayed demixing process. Through this process, demixing required a longer time and the exchange between the solvent and the nonsolvent occurred at a slow rate after the immersion. This has resulted in the formation of sponge-like mesoporous polymer network [53,54].

For DL-ZT0, the SEM images showed good adherence between the inner and outer layers with no sign of delamination at an air gap of 5, 10, and 20 cm. The two layers are compatible as they comprised of the same polymer. As the air gap length was increased to 30 cm, the outer layer formed a dense layer above the finger-like structure. The thicker outer layer was influenced by the lower concentration of the outer layer dope solution extruded at double the speed of the inner layer dope solution. Delamination of the inner and outer layer became visible at an air gap of 30 cm and significant at air gap length of 40 to 50 cm with the clear appearance of macrovoids around the finger-like at the inner layer. The delamination between the outer and inner layers disrupted the membrane structure.

**Figure 8:**
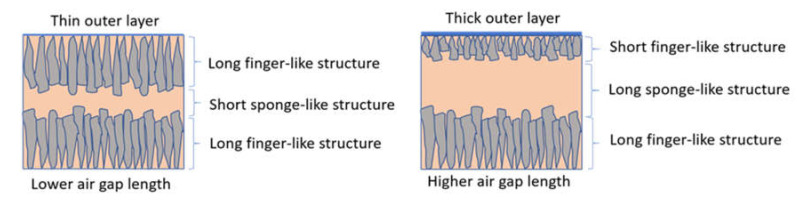
Schematic illustration of the effect of air gap on DLHF membrane structure.

However, in DL-ZT1, no sign of delamination was observed throughout the air gap range of 5–50 cm, indicating a better morphological structure of DLHF membranes embedded with ZrO_2_-TiO_2_ nanoparticles. This can be explained by the higher shrinkage rate of the outer layer than the inner layer during the exchange process between the solvent and the nonsolvent. The added ZrO_2_-TiO_2_ nanoparticles resulted in the formation of microvoids at the outer layer which exhibited a higher shrinkage rate [55]. This caused the membrane inner layer to be firmly encircled by the membrane outer layer and produced a continuous interface between the layers as reported elsewhere [52]. The schematic diagram in Figure 8 depicts the effect of air gap length on the outer layer thickness and the size of the finger-like and sponge-like structure.

### 3.4. Effect of Air Gap on Membrane Hydrophilicity

**Figure 9:**
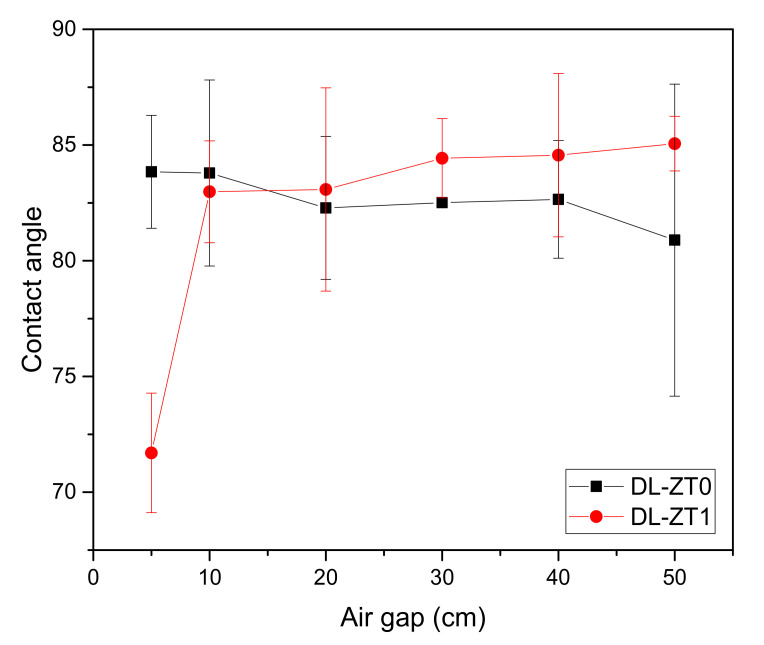
Contact angle measurement with increasing air gap length.

The plots of contact angle measurement with increasing air gap length are shown in Figure 9. The contact angle for DL-ZT0 showed a slight reduction from 83.84° ± 2.44° to 80.89° ± 6.74° for the air gap of 5 to 50 cm. Meanwhile, the contact angle for DL-ZT1 showed increment from 71.70° ± 2.58° to 85.06° ± 1.18°. Interestingly, the contact angle of DL-ZT1 spun at 5 cm recorded the lowest reading.

Reduction in the contact angle reading showed the advantage of spinning the fiber at a lower air gap which helped in the improvement of wetting phenomena. The surface hydrophilicity increased when more nanoparticles were concentrated at the outer layer during the membrane formation stage [56]. The addition of ZrO_2_-TiO_2_ nanoparticles was believed to introduce a substantial amount of hydroxyl radicals, which in turn improves the hydrophilicity of the membrane. However, the contact angle of DL-ZT1 started to increase when spun at an air gap above 10 cm. Larger air gap resulted in the reduction in the outer radial dimension which disrupted the membrane wetting and decreased the hydrophilicity of the membrane. Within the experimental error, no significant change in the contact angle was observed when the air gap was increased from 10 cm to 50 cm. This may be caused by the stable deposition of sessile drops on the specimen surface during contact angle measurement [57]. On another note, the huge deviation in the contact angle at an air gap of 50 cm in DL-ZT1 might be due to the surface heterogeneity of the membrane that could cause variations of the contact point along the three-phase contact line [58].

### 3.5. Effect of Air Gap on Membrane Porosity

Table 2 showed the results of membrane porosity with an increasing air gap. Both DL-ZT0 and DL-ZT1 membranes showed an increasing porosity trend. However, the increase in porosity was more apparent in DL-ZT0 compared to DL-ZT1. The addition of ZrO_2_-TiO_2_ nanoparticles into the polymer matrix hindered the phase inversion process and reduced the porosity of the membrane. The improvement in the membrane porosity of both membranes can be explained as the effect of gravitational pull at higher air gap towards the membrane structure which has in turn produced a bigger pore size compared to that of fabricated at lower air gap. Table 2 summarizes the results of porosity for both DL-ZT0 and DL-ZT1 membranes. The sufficiently strong elongation stress during spinning has pulled the polymer molecular chains apart at the beginning of phase separation and created a porous structure which resulted in the increase of the membrane free volume [59]. On the other hand, the weak stress exerted on the membrane spun at the air gap of 5 cm has induced the unfavorable change in the molecular orientation which reduced membrane porosity [35].

### 3.6. Effect of Air Gap on Membrane Performance

**Figure 10:**
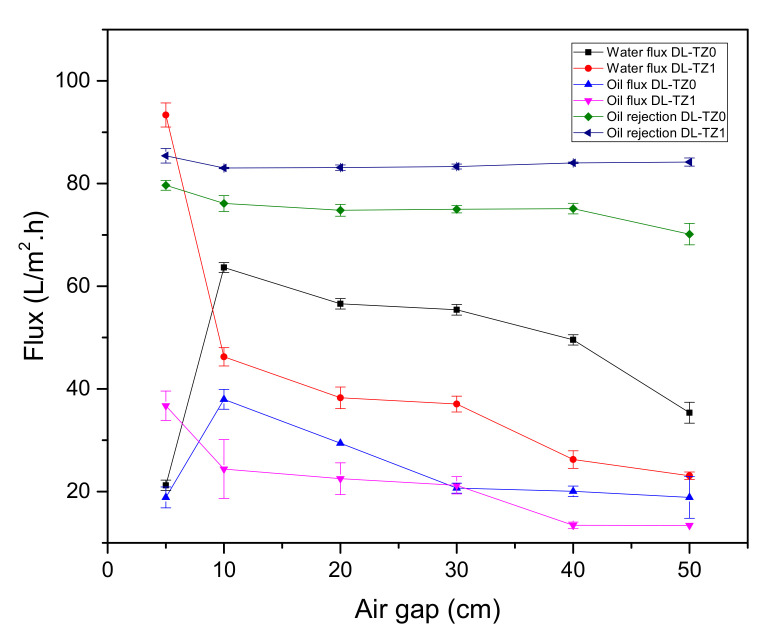
Effect of air gap on flux and oil rejection.

Figure 10 shows the performance of DL-ZT0 and DL-ZT1 spun at a different air gap for oily wastewater treatment. The water flux of DL-ZT0-05, DL-ZT0-10, DL-ZT0-20, DL-ZT0-30, DL-ZT0-40, and DL-ZT0-50 was 21.2, 63.7, 56.6, 55.4, 49.5, and 35.4 L/m^2^.h, respectively. Meanwhile, the water flux of DL-ZT1-05, DL-ZT1-10, DL-ZT1-20, DL-ZT1-30, DL-ZT1-40, and DL-ZT1-50 was 93.4, 46.2, 38.3, 37.0, 26.2, and 23.1 L/m^2^.h, respectively. The water flux of DL-ZT1-05 recorded the highest reading and improved by about 341% from the water flux of DL-ZT0-05 before exhibited the decreasing trend. The same trend was observed in the oily wastewater flux but at a much lower reading. The oily wastewater flux of DL-ZT0-05, DL-ZT0-10, DL-ZT0-20, DL-ZT0-30, DL-ZT0-40, and DL-ZT0-50 was 18.9, 38.0, 29.4, 20.7, 20.0, and 18.9 L/m^2^.h, respectively. On the other hand, the oily wastewater flux of DL-ZT1-05, DL-ZT1-10, DL-ZT1-20, DL-ZT1-30, DL-ZT1-40, and DL-ZT1-50 was 36.7, 24.4, 22.5, 21.2, 13.5, and 13.4 L/m^2^.h, respectively. The result showed that the oily wastewater flux of DL-ZT1-05 recorded the highest reading and surpassed the oily wastewater flux of DL-ZT0-05 by 94.2% before dropping further as the air gap increased.

The increase in pure water flux in DL-ZT1-05 was caused by the longer finger-like structure on the outer surface, small area of sponge-like structure as well as the expansion of the inner layer diameter dimensions of the membrane as discussed in Sec. 3.3. Therefore, the water flux increases. The uniformly distributed ZrO_2_-TiO_2_ nanoparticles on the membrane outer layer enhanced the hydrophilicity of the membranes and improved the oil rejection percentage. Oil rejection percentage of 85.4% was recorded for DL-ZT1-05 compared to 79.7% in DL-ZT0-05. The reduction in both water and oily wastewater fluxes with increasing air gap could be ascribed to the extra elongated air gap length which has caused phase instability in the membrane. The formation of non–selective voids in the membrane reduced the oil rejection percentage. In this work, the highest oil rejection was exhibited by the DLHF membrane embedded with ZrO_2_-TiO_2_ nanoparticles spun at the air gap of 5 cm. The rejection was 5.7% higher than that of the neat membrane.

## 4. Conclusions

In the current work, the effect of air gap was optimized to fabricate PVDF DLHF membranes incorporated with ZrO_2_-TiO_2_ nanoparticles for oily wastewater treatment. TEM and EDX analysis revealed that the ZrO_2_-TiO_2_ nanoparticles having an average grain size of ~12 nm and the Ti, Zr and O elements were uniformly distributed throughout the sample. In relation to the effect of air gap, SEM images of the PVDF DLHF membranes showed the development of a finger-like structure at both inner and outer layers. The increment of air gap caused the outer layer to became denser while the finger-like structure become smaller and shorter. These results were mainly because of the longer contact time between the spun fiber with the air medium first before contacting with water which caused the surface of the outer layer to solidify in air during traveling. The permeation results revealed that the water flux of DL-ZT1 was enhanced and the oil rejection was recorded as 85.4% when the membrane was spun at an optimum air gap of 5 cm. This result was supported by the SEM images which showed the longest finger-like structure formed at the outer layer just below the selective layer produced as well as a smaller area of a sponge-like structure. The enhancement in the oil rejection demonstrated the potential of ZrO_2_-TiO_2_ incorporated PVDF DLHF to be used for oily wastewater treatment.

## Figures and Tables

**Table 1:** Spinning conditions for PVDF/ZrO_2_-TiO_2_ DLHF membrane fabrication.

Parameters	Values
Outer dope composition (wt%)	PVDF/ZrO_2_-TiO_2_/NMP/PVP (15/1/80/5)
Inner dope composition (wt%)	PVDF/NMP/PVP (18/77/5)
Outer dope flowrate (cm^3^/min)	2.48
Inner dope flowrate (cm^3^/min)	1.2
Bore fluid	Distilled water
Bore fluid flowrate (cm^3^/min)	0.73
Air gap (cm)	5
Take up speed (cm/s)	Free fall
Spinneret dimension	1.2/0.8/0.4

**Table 2:** Results of porosity for DL-ZT0 and DL-ZT1 at different air gaps.

Samples	Porosity	Samples	Porosity
DL-ZT0–5	49.7	DL-ZT1–5	45.5
DL-ZT0–10	50.9	DL-ZT1–10	46.3
DL-ZT0–20	60.0	DL-ZT1–20	47.2
DL-ZT0–30	58.5	DL-ZT1–30	46.9
DL-ZT0–40	56.9	DL-ZT1–40	47.0
DL-ZT0–50	58.1	DL-ZT1–50	46.7
